# Supplementary material for: Functional brain network reconfiguration during learning in a dynamic environment
Source: Nat Commun. 2020 Apr 3;11:1682. doi: 10.1038/s41467-020-15442-2 (PMC7125157; doi:10.1038/s41467-020-15442-2)
Supplement: Supplementary file 3 — Reporting Summary [file 41467_2020_15442_MOESM3_ESM.pdf]

nature research

Corresponding author(s): Joseph W. Kable

Last updated by author(s): Feb 27, 2020

Reporting Summary

Nature Research wishes to improve the reproducibility of the work that we publish. This form provides structure for consistency and transparency in reporting. For further information on Nature Research policies, see Authors & Referees and the Editorial Policy Checklist .

Statistics

For all statistical analyses, confirm that the following items are present in the figure legend, table legend, main text, or Methods section.

n/a	Confirmed	
☐	☒	The exact sample size (*n*) for each experimental group/condition, given as a discrete number and unit of measurement
☐	☒	A statement on whether measurements were taken from distinct samples or whether the same sample was measured repeatedly
☐	☒	The statistical test(s) used AND whether they are one- or two-sided<br>*Only common tests should be described solely by name; describe more complex techniques in the Methods section.*
☐	☒	A description of all covariates tested
☐	☒	A description of any assumptions or corrections, such as tests of normality and adjustment for multiple comparisons
☐	☒	A full description of the statistical parameters including central tendency (e.g. means) or other basic estimates (e.g. regression coefficient) AND variation (e.g. standard deviation) or associated estimates of uncertainty (e.g. confidence intervals)
☐	☒	For null hypothesis testing, the test statistic (e.g. *F*, *t*, *r*) with confidence intervals, effect sizes, degrees of freedom and *P* value noted<br>*Give P values as exact values whenever suitable.*
☒	☐	For Bayesian analysis, information on the choice of priors and Markov chain Monte Carlo settings
☒	☐	For hierarchical and complex designs, identification of the appropriate level for tests and full reporting of outcomes
☐	☒	Estimates of effect sizes (e.g. Cohen's *d*, Pearson's *r*), indicating how they were calculated

Our web collection on statistics for biologists contains articles on many of the points above.

Software and code

Policy information about availability of computer code

Data collection	Data were collected in a published study (McGuire et al., 2014, Neuron), in which the experiment task was implemented in Matlab using MGL (http://justingardner.net/mgl) and SnowDots (http://code.google.com/p/snow-dots) extensions.
Data analysis	Custom codes established in Python 2.7 were used to implemented non-negative matrix factorization (https://github.com/akhambhati/Echobase). Matlab was used to implement behavior modeling, statistical testing and visualization (https://github.com/changhaokao/nmf_network_learning).

For manuscripts utilizing custom algorithms or software that are central to the research but not yet described in published literature, software must be made available to editors/reviewers. We strongly encourage code deposition in a community repository (e.g. GitHub). See the Nature Research guidelines for submitting code & software for further information.

Data

Policy information about availability of data

All manuscripts must include a data availability statement. This statement should provide the following information, where applicable:

- Accession codes, unique identifiers, or web links for publicly available datasets
- A list of figures that have associated raw data
- A description of any restrictions on data availability

The data for the current study are available from the corresponding author upon request.

Field-specific reporting

Please select the one below that is the best fit for your research. If you are not sure, read the appropriate sections before making your selection.

Life sciences study design

All studies must disclose on these points even when the disclosure is negative.

Sample size	The current study used data from a published study (McGuire et al., 2014, Neuron), in which the number of sample size is 32. This sample size have shown to be sufficient to investigate participants' learning performance (e.g., Nassar et al., 2012, Nature Neuroscience) and examined dynamic functional connectivity with non-negative matrix factorization (e.g., Khambhati et al., 2018, PLoS Computational Biology).
Data exclusions	No data were excluded.
Replication	We investigated functional connectivity in 25-second sliding windows in the main analyses and we also examined the robustness of our results in shorter sliding time windows (20 seconds) and longer sliding time windows (30 seconds). All the results were replicated in the shorted and longer time windows while the effects were weaker in the shorter time windows though.
Randomization	No randomization of participants were implemented since we implemented the within-subject design. All participants performed the task in the same conditions.
Blinding	Blinding to the conditions was not necessary since all participants performed the task in the same conditions. In addition, subjects were instructed about the current condition (i.e., noise level) at the beginning of each run.

Reporting for specific materials, systems and methods

We require information from authors about some types of materials, experimental systems and methods used in many studies. Here, indicate whether each material, system or method listed is relevant to your study. If you are not sure if a list item applies to your research, read the appropriate section before selecting a response.

Materials & experimental systems

n/a	Involved in the study	
☒	☐	Antibodies
☒	☐	Eukaryotic cell lines
☒	☐	Palaeontology
☒	☐	Animals and other organisms
☐	☒	Human research participants
☒	☐	Clinical data

Methods

n/a	Involved in the study	
☒	☐	ChIP-seq
☒	☐	Flow cytometry
☐	☒	MRI-based neuroimaging

Human research participants

Policy information about studies involving human research participants

Population characteristics	Thirty-two individuals participated in the fMRI experiment: 17 females, mean age = 22.4 years (SD = 3.0; range 18-30).
Recruitment	Participants were recruited from the University of Pennsylvania community. Participants should be between 18 and 30 years old, had no history of neurological or psychological disorders and were not currently under any medication.
Ethics oversight	Internal Review Board in University of Pennsylvania

Note that full information on the approval of the study protocol must also be provided in the manuscript.

Magnetic resonance imaging

Experimental design

Design type	task; event-related design.
Design specifications	Participants performed four 120-trials runs during functional scanning. On each trial, participants made a prediction within 3 seconds and then outcome was shown for 1.6 seconds. The next prediction immediately followed the presentation of the current outcome.
Behavioral performance measures	Participants' prediction on each trial were collected. A multiple regression was conducted to investigate how participants' update was influenced by different factors (e.g., surprise, uncertainty and reward).

Acquisition

Imaging type(s)	functional
Field strength	3T
Sequence & imaging parameters	Functional data were acquired using a gradient-echo echoplanar imaging (EPI) (3 mm isotropic voxels, 64 x 64 matrix, 42 axial slices tilted 30 degree from the AC-PC plane, TE = 25 ms, flip angle = 75 degree, TR = 2500 ms). There were four runs with 226 images per run. T1-weighted MPRAGE structural images (0.9375 X 0.9375 X 1 mm voxels, 192 X 256 matrix, 160 axial slices, TI = 1100 ms, TE = 3.11 ms, flip angle = 15 degree, TR = 1630 ms) and matched fieldmap images (TE = 2.69 and 5.27 ms, flip angle = 60 degree, TR = 1000 ms) were also collected.
Area of acquisition	a whole brain scan

Diffusion MRI ☐ Used ☒ Not used

Preprocessing

Preprocessing software	FSL and AFNI were used to preprocess data. Functional data were temporally aligned to the midpoint of each volume (AFNI's 3dTshift), motion corrected (FSL's MCFLIRT), registered to a MNI template (FSL's FLIRT and FNIRT), outlier-attenuated (AFNI's 3dDespike), spatial smoothed with a 6 mm FWHM Gaussian kernel (FSL's fslmaths) and intensity-scaled by a single grand mean in each run. Structure images were brain extracted, registered to a MNI template (FSL's FLIRT and FNIRT) and segmented into gray matter, white matter (WM) and cerebrospinal fluid (CSF) (FSL's FAST).
Normalization	Both functional and structural images were registered and normalized to the standard MNI space. Functional data were first aligned to the structural image using boundary-based registration and fieldmap-based geometric undistortion was also incorporated. In addition, structural image was nonlinearly coregistered to the MNI template. Both transformation were applied to the functional data.
Normalization template	MNI152
Noise and artifact removal	Before calculating functional connectivity between ROIs, time series of each ROI were band-pass filtered with the cutoff of 0.01-0.08 Hz. Then, for each time series, a confound regression was applied. This confound regression included 24 motion parameters (three translation and three rotation motion parameters and their expansion ([R_t R_t^2 R_(t-1) R_(t-1)^2])54, as well as average signals from WM and CSF.
Volume censoring	To ensure magnetization equilibrium, the first 6 volumes of each run were removed from the analysis.

Statistical modeling & inference

Model type and settings	For one analysis, we estimated mass univariate regression coefficients of our interested task variables at the first level (within each run and participant) using the fixed effects model.
Effect(s) tested	We did not tested the effects on these univariate regression coefficients. Instead, these regression coefficients were used to re-constructed predicted time series for task variables for each run and participant, and then we used these predicted time series in the functional connectivity analysis.

Specify type of analysis: ☐ Whole brain ☒ ROI-based ☐ Both

Anatomical location(s)	For the following functional connectivity analyses, we focused on ROIs from the parcellation in Power et al. (2011, Neuron). There were 264 ROIs but we kept 247 ROIs which had usable data for all participants.
Statistic type for inference (See Eklund et al. 2016)	No inference was made for the above model fitting.
Correction	n/a

Models & analysis

n/a	Involved in the study
☐	☒ Functional and/or effective connectivity
☐	☒ Graph analysis
☒	☐ Multivariate modeling or predictive analysis

Functional and/or effective connectivity	Pearson correlation coefficients between every pairs of ROIs were used as functional connectivity.
Graph analysis	We used the non-negative matrix factorization to decompose whole-brain dynamic functional connectivity into specific patterns of functional connectivity, called subgraphs, and the expression of these patterns over time. We formed this whole-brain dynamic functional connectivity by concatenating the matrix of functional connectivity from all subjects. The pattern of functional connectivity in each subgraph was then summarized as the strength within the same functional system and the strength between different functional systems.
